# The persuasiveness of vaccine focused social media posts to parents of adolescents

**DOI:** 10.1016/j.pmedr.2025.103194

**Published:** 2025-08-05

**Authors:** Teresa M. Imburgia, Holly B. Fontenot, Gary H.R. Glauberman, Erica Liebermann, Melanie L. Kornides, Eunjung Lim, Masako Matsunaga, Gregory D. Zimet

**Affiliations:** a Indiana University Richard M. Fairbanks School of Public Health & Melvin & Bren Simon Comprehensive Cancer Center, 550 University Blvd., AOC 6046, Indianapolis, IN 46202, USA; b School of Nursing, University of Hawaii at Manoa, 2528 McCarthy Mall, Webster Hall 402, Honolulu, HI 96822, USA; c College of Nursing, University of Rhode Island, Providence, 350 Eddy Street Rm 223, Providence, RI 02903, USA; d University of Pennsylvania School of Nursing, 418 Curie Blvd, Philadelphia, PA 19104, USA; e Department of Quantitative Health Sciences John A. Burns School of Medicine, 651 Ilalo Street, Honolulu, HI 96813, USA; f Department of Pediatrics, Indiana University School of Medicine, 410 W. 10th Street, HS, 1001, Indianapolis, IN, USA

## Abstract

**Objective:**

Social media and online health information play an important role in vaccine decision making. Our aim is to examine the persuasiveness of social media posts about vaccines for mothers of youth by vaccine hesitancy.

**Methods:**

A U.S. national survey of mothers of adolescents aged nine to 17 in 2023 assessed persuasiveness of vaccine-related social media posts. Persuasiveness dimensions included information source, presence of a hyperlink, vaccine type, and message type. A fractional-factorial design identified nine posts/scenarios, varying across the four dimensions. Respondents rated the persuasiveness of each from zero to 100. Ratings-based conjoint analysis evaluated relative preferences, translated into importance scores, indicating the influence of each stratified by low, medium, and high vaccine hesitancy.

**Results:**

The 3803 mothers indicated that information source (American Academy of Pediatrics [AAP] most preferred; importance score = 57.2) contributed most strongly to persuasiveness, followed by vaccine type (human papillomavirus [HPV] most preferred; importance score = 22.9), then preference for a hyperlink (importance score = 17.1), and a statistical post (importance score = 2.8). Stratified by vaccine hesitancy, the mean persuasiveness across all nine posts/scenarios was significantly different between the groups. All groups reported information source (AAP most preferred) and vaccine type (HPV most preferred) as most persuasive. All preferred hyperlinks, but the higher hesitancy groups found narrative posts more persuasive.

**Conclusions:**

These data highlight the importance of posts directed at parents emanating from trusted sources like the AAP, with hyperlinks to further information. For individuals with vaccine hesitancy, our findings support sharing stories instead of statistical posts.

## Highlights

• Social media posts on vaccination directed should come from trusted sources.
• Hyperlinks to further information increase the persuasiveness of the messages.
• For vaccine hesitant parents, sharing stories is more persuasive.

## 1 Introduction

The spread of misinformation is a public health crisis and has negatively impacted vaccination coverage (Merchant et al., 2021). Vaccines have been the gold standard of disease prevention, yet public trust in vaccines has declined, in part, due to social media misinformation (Lazarus et al., 2024). Worldwide, more than 4.26 billion people utilize social media, and many people use it as a primary source of health information (Jafar et al., 2023). This has had both positive and negative effects. It has the power to communicate quickly with large numbers of people, but it can also spread health misinformation quickly (Merchant et al., 2021) and across multiple platforms (Boatman et al., 2024).

Both pro- and anti-vaccination posts may impact vaccination rates (Ortiz et al., 2019). The impact of social media is one factor in several complex and interacting factors that can impact vaccination rates. There are limited studies examining social media interventions and message attributes. A recent review found that over the last decade a wide variety of interventions have been implemented on social media to increase vaccine knowledge, attitudes, intentions, and behaviors with varied results (Limaye et al., 2021). Those authors called the field of social media and vaccine hesitancy nascent and stated that more strategies are needed (Limaye et al., 2021). The studies included in the review did not measure what about those interventions, i.e. the dimensions of the posts were most compelling, nor did they examine the difference in intervention effectiveness between varying levels of vaccine hesitancy.

Vaccine hesitancy encompasses a range of attitudes and beliefs associated with vaccine decision-making, and can lead to increases in vaccine refusal or delay (MacDonald, 2015). Simple pro-vaccine messaging is often ineffective and can backfire, decreasing the intention to vaccinate, especially for those who are already vaccine hesitant (Unicef, 2020). Few interventions have focused on messaging interventions for those with high vaccine hesitancy (Dubé et al., 2015). A recent assessment of vaccine hesitancy in the United States (U.S.) estimated that 6 % to 25 % of parents may be vaccine hesitant, that hesitancy is higher for influenza and human papillomavirus (HPV) vaccines, and addressing parent hesitancy has become more challenging (Nowak and Cacciatore, 2023). Since the availability of the COVID-19 vaccine, vaccine hesitancy among parents of children aged five to 11 years increased from 19.8 % to 21.0 % from 2019 to 2022 (Vashist et al., 2024).

Lacking in the literature are what dimensions or characteristics of posts are most persuasive to parents. In a recent review of social media strategies to affect vaccine acceptance, the interventions varied widely using a multitude of strategies to target one specific population (Limaye et al., 2021). In short, we still do not know what specific aspects of posts implemented in interventions are most persuasive to change vaccine behavior. In this study we aimed to identify the most persuasive dimensions of social media posts directed at mothers of adolescents to increase vaccine acceptability by vaccine hesitancy levels.

## 2 Methods

### 2.1 Study sample

A U.S. national survey of mothers of adolescents aged nine to 17, fielded in July 2023, was used to assess the dimensions of persuasiveness of vaccine-related social media posts. Potential participants were recruited and managed by Dynata™, an experienced marketing research company. This allowed researchers to access quality large samples for online quantitative studies in a timely manner. Participants were contacted by Dynata and if interested they were provided additional study information. If the participants were willing to participate, they were sent an online consent and survey via Qualtrics. Eligibility criteria included: 1) mother or identified female primary caregiver of an adolescent aged nine to 17 years, 2) ability to read English, 3) access to a computer/tablet with internet access, and 4) utilization of at least one form of social media. The University of Hawai'i at Manoa Institutional Review Board approved this study.

### 2.2 Measures

**Fig. 1:**
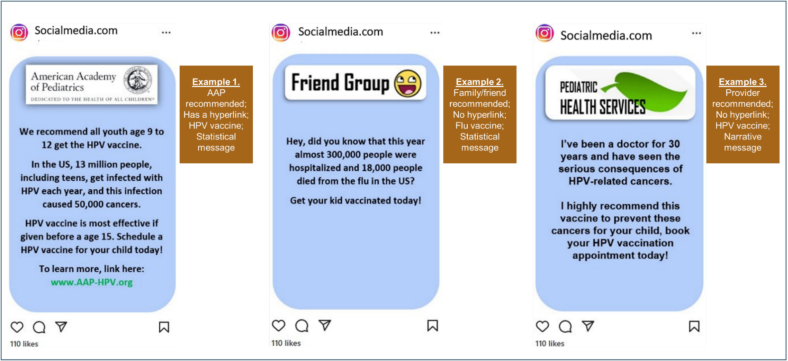
Example social media post scenarios presented to 3968 U.S. mothers of adolescents, July 2023.

To assess the persuasiveness of the social media posts, participants responded to nine items. These were presented in random order by the survey to eliminate ordering effects. A fractional-factorial design identified nine posts/scenarios, each varying across the four dimensions. Respondents rated the persuasiveness of each post/scenario on a scale from zero (not at all persuasive) to 100 (completely persuasive). The dimensions were: information source (American Academy of Pediatrics [AAP], health care provider [provider], or friends/family), presence of a hyperlink (yes, no), vaccine type (HPV, Influenza [Flu], or general adolescent vaccines), and message type (narrative, statistical). The posts were created by the study team informed by both literature and by the findings of online focus groups of parents (Fontenot et al., 2024). Examples of the posts can be seen in Fig. 1.

We additionally collected sample demographics and measured vaccine hesitancy. Demographics included age, race/ethnicity, number of children, level of education, neighborhood (rural vs. urban), health insurance status, and use of social media. Use of social media was captured on a 6-point Likert-type frequency scale from daily use to no use of a particular platform.(Liebermann et al., 2025) The General Vaccine Hesitancy Scale (α = 0.831) consisted of six items (example item: c*hildhood vaccines are generally safe*) measured on a five-point Likert type scale from strongly disagree to strongly agree (Helmkamp et al., 2021).

### 2.3 Statistical analysis

We provided descriptive statistics on the sample along with the difference in the mean persuasiveness of posts by vaccine hesitancy groups using ANOVA with a post-hoc Tukey test. The vaccine hesitancy score was calculated using the mean of the six items in the scale. We then stratified the sample low, medium, and high hesitancy groups pegged to the scale anchors of strongly disagree with vaccine hesitant statements (1) to strongly agree with vaccine hesitant statements (5). This was done to provide clear delineation of low vs high hesitancy since the distribution was skewed towards lower hesitancy. Low hesitancy consisted of any score under 2.5, medium hesitancy consisted of those who scored 2.5–3.4, and the high hesitancy group was anyone who scored 3.5 and above.

Full-profile ratings-based conjoint analysis was used to evaluate the effects of social media post characteristics on parental perceptions of the persuasiveness of the posts. This regression-based analytic technique is frequently used in marketing research to evaluate how the characteristics of a product influence the product's acceptability (Churchill and Iacobucci, 2006). This analysis can only be performed with individuals who showed variability across the nine posts. Those without variabilities across the nine posts (e.g., anyone who rated all of the scenarios with a score of 100) exhibited no relative preferences and are therefore not included in the conjoint analysis. Ratings-based conjoint analysis evaluated relative preferences (i.e., part-worth utilities). The range of the part-worth utilities was translated into importance scores, indicating the influence of each dimension on persuasiveness.

We compared the perceived persuasiveness of posts across the groups and performed conjoint analysis separately for each of the hesitancy groups. All analyses were conducted using SPSS (IBM Corp, 2026), with statistical significance defined as a *p*-value less than 0.05.

## 3 Results

The sample consisted of 3968 mothers with at least one youth aged nine to 17. Almost half (48.8 %) were 35–44 years old, 73.7 % were white, 40.1 % had some college or associated degree, and 51.9 % were from suburban neighborhoods. Stratified on General Vaccine Hesitancy scores, 57.4 % had low hesitancy, 34.0 % had medium hesitancy, and 8.6 % had high vaccine hesitancy. Full demographics are listed in Table 1.

**Table 1:** Characteristics of 3968 parents of adolescents: United States, July 2023.

Variables	n; % or mean (SD)
*Age*	
24 years or younger	35; 0.9 %
25–34 years	714; 18.0 %
35–44 years	1937; 48.8 %
45–54 years	1010; 25.5 %
55–64 years	220; 5.5 %
65 years or older	52; 1.3 %

*Race*	
Asian	111; 2.8 %
Black	431; 10.9 %
Hispanic	339. 8.5 %
Mixed/More than one	90; 2.3 %
Native American, American Indian, or Alaska native	50; 1.3 %
Native Hawaiian or another Pacific Islander identity	82 0.2 %
Something else	15; 0.4 %
White	2924; 73.7 %

*Number of children 9–17*	
1	2171; 54.7 %
2	1331; 33.5 %
3	352; 8.9 %
4	88; 2.2 %
5 or more	26; 0.7 %

*Highest level of education*	
High school, GED, or less	937; 23.6 %
Some college or associated degree	1590; 40.1 %
Bachelor's degree	932; 23.5 %
Graduate degree	509; 12.8 %

*Neighborhood*	
Rural	1045; 26.3 %
Suburban	2059; 51.9 %
Urban	864; 21.8 %

*Child's health insurance*	
Private	2052; 51.7 %
Public	1779; 44.8 %
Not sure/ don't know	137; 3.5 %

*Daily use of:*	
Facebook	2958; 74.5 %
Instagram	1615; 40.7 %
TikTok	1336; 33.7 %
Twitter	379; 9.6 %

*General Vaccine Hesitancy Scale, Range (1–5)*	2.4 (0.3)
Low	2277; 57.4 % | 2.0 (0.3)
Medium	1350; 34.0 % | 2.7 (0.3)
High	341; 8.6 % | 3.7 (0.5)

*Persuasiveness rating, Range (0−100)*	
Overall	50.1 (27.0)
By General Vaccine Hesitancy scale^a^	
Low	59.1 (24.2)
Medium	41.3 (24.9)
High	25.2 (26.1)

a. ANOVA between groups (*F* = 411.6, p < .001). In post-hoc Tukey, persuasiveness of posts for the low vaccine group was significantly higher than the medium group (p < .001), which was also significantly higher than the high hesitancy group (p < .001).

The mean persuasiveness rating across the nine posts for the sample was 50.1 (standard deviation [SD] = 27.0). When stratified by HPV vaccine status, persuasiveness differences emerged between those who were in the low, medium, and high vaccine hesitancy groups. ANOVA revealed a significant difference in the mean persuasiveness rating across all nine posts/scenarios for the different hesitancy groups (F = 411.6; *p* < .001). In post-hoc analysis, the low hesitancy group had a significantly higher persuasiveness mean (59.1, SD = 24.2) than the medium hesitancy group (41.3, SD = 26.1), which also had a significantly higher mean than the high hesitancy group (25.2, SD = 26.1).

**Fig. 2:**
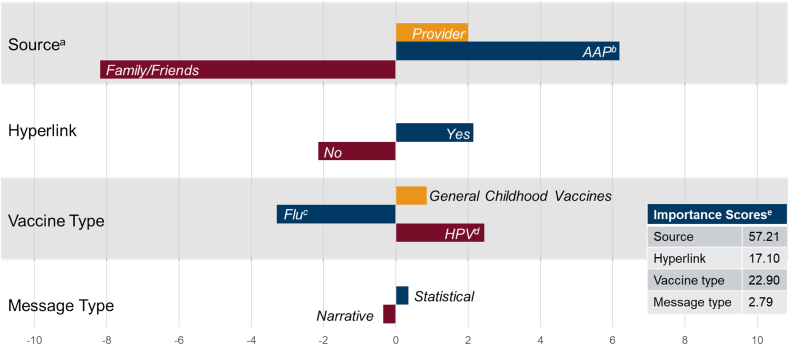
Overall Part-Worth Utility Scores and importance scores of persuasiveness of vaccine related social media posts of 3803 mothers of adolescents: United States, July 2023. Note. 3803 / 3968 (95.8 %) showed variability across persuasiveness ratings m = 50.1 (SD = 27.0). Of the 165 parents that did not change persuasiveness ratings across social media posts, 55.8 % rated persuasiveness of all posts zero and 38.2 % rated persuasiveness of all posts 100. a. The part-worth utility score here represents the overall relative preference that the participating mothers of adolescents placed on each level of an attribute. b. AAP = American Academy of Pediatrics. c. Flu = Influenza vaccine. d. HPV = Human papillomavirus vaccine. e. The importance score reflects how significant the attribute is relative to others in shaping preferences, expressed as a percentage.

Of the 3803 (95.8 % of the total sample) who showed variability across persuasiveness ratings, conjoint modeling reveled that the dimension that contributed most strongly to persuasiveness was information source (AAP most preferred; importance score = 57.2), followed by preference of having a hyperlink (importance score = 22.9), vaccine type (HPV most preferred; importance score = 17.1) and a statistical message (importance score = 2.8) (Fig. 2). For those without variability across scores, 55.8 % rated all posts at zero persuasiveness and 38.2 % rated all posts at 100.

**Fig. 3:**
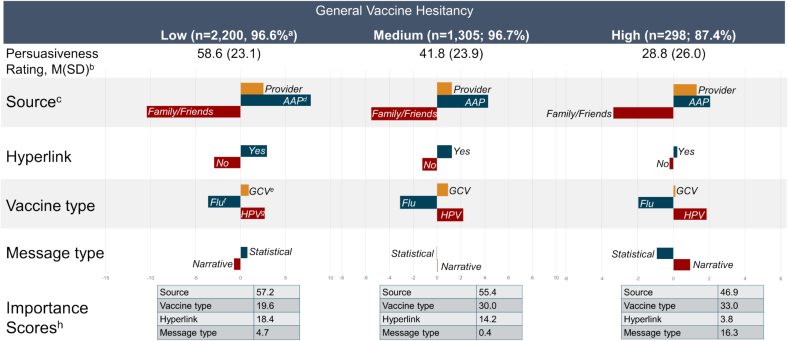
Part-Worth Utility Scores and importance scores on persuasiveness of vaccine related social media posts of 3803 mothers of adolescents stratified by vaccine hesitancy: United States, July 2023. Note. There are differences in scaling along the x-axis for each hesitancy group. a. The percentage for each low, medium, and high hesitancy reflects the percentage of those in each group that had variability in persuasiveness ratings. b. Mean (Standard Deviation) of each group. ANOVA of persuasiveness scores by hesitancy group F = 411.6; *p* < .001. c. The part-worth utility score here represents the overall relative preference that the participating mothers of adolescents placed on each level of an attribute. d. AAP = American Academy of Pediatrics. e. GCV = General childhood vaccines f. Flu = Influenza vaccine. g. HPV = Human papillomavirus vaccine h. The importance scores reflect how significant the attribute is relative to others in shaping preferences, expressed as a percentage for each hesitancy group.

For the low vaccine hesitancy group, 2200 out of 2277 (96.6 %) showed variability across persuasiveness ratings, and had a mean persuasiveness rating of 58.6 (SD = 23.1). Conjoint modeling reveled that the dimension that contributed most strongly to persuasiveness was information source (AAP most preferred; importance score = 57.2), followed by vaccine type (HPV most preferred; importance score = 19.6), preference of having a hyperlink (importance score = 18.4), and a statistical message (importance score = 4.7) (Fig. 3). For those without variability across scores, 26.0 % rated all posts at zero persuasiveness, and 68.8 % rated all posts at 100.

For the medium vaccine hesitancy group, 1305 out of 1350 (96.7 %) showed variability across persuasiveness ratings and had a mean persuasiveness rating of 41.8 (SD = 23.9). Conjoint modeling reveled that the dimension that contributed most strongly to persuasiveness was information source (AAP most preferred; importance score = 55.4), followed by vaccine type (HPV most preferred; importance score = 30.0), preference of having a hyperlink (importance score = 14.2), and a narrative message (importance score = 0.4) (Fig. 3). For those without variability across scores, 68.9 % rated all posts at zero persuasiveness, and 22.2 % rated all posts at 100.

For the high vaccine hesitancy group, 298 out of 341 (87.4 %) showed variability across persuasiveness ratings, with a mean persuasiveness rating of 28.8 (SD = 26.0). Respondents indicated that the dimension that contributed most strongly to persuasiveness was information source (AAP most preferred; importance score = 46.9), followed by vaccine type (HPV most preferred; importance score = 33.0), a narrative message (importance score = 16.3), and preference of having a hyperlink (importance score = 3.8) (Fig. 3). For those without variability across scores, 95.3 % rated all posts at zero persuasiveness, and 4.7 % rated all posts at a 100.

## 4 Discussion

Overall persuasiveness of our posts was around 50 % and was inversely associated with vaccine hesitancy, adding validity to the hesitancy score (Szilagyi et al., 2020). Over 90 % of the sample was either low or medium hesitancy, signaling that most people are open to vaccination and that tools such as clinicians using a presumptive recommendation approach and/or motivational interviewing techniques may be effective for many parents (Dempsey et al., 2018; Opel et al., 2013). However, these scores point to the difficulty of reaching high hesitancy parents with any vaccine promoting social media posts. The built-in resistance is a huge challenge to overcome.

The overall conjoint provided relative preferences for AAP as the source, the presence of a hyperlink, and a focus on HPV vaccination. There was a preference for statistical posts, but the importance score of that was low in the overall group. The source of the information regardless of parent vaccine hesitancy was the most important component of the persuasiveness of the posts. This is similar to what parents have reported in the clinical setting that vaccine uptake is very reliant on the clinician's recommendation (Lin et al., 2021; Oh et al., 2021). Social media posts focused on vaccines should come from reliable, trusted sources.

The low and medium hesitancy groups were similar in their first three preferences with the source (AAP preferred) as most important followed by vaccine type (HPV most preferred), then the preference for a hyperlink. However, differences were observed when comparing the low and medium hesitancy groups. The importance of message type was higher in the low hesitancy group (4.7 vs 0.4) compared to the medium hesitancy group, with a preference for statistical posts in the low hesitancy group.

When we analyzed the high hesitancy group, they were similar to the low and medium hesitancy groups in their rating of the source (AAP most preferred for all groups) as the most important though the importance score gradually declined from low (57.2), medium (55.4), and high (46.9) hesitancy groups. Second most important to all groups was the HPV for vaccine type in all groups with the importance increasing from the low (19.6), medium (30.0), and high (33.0) hesitancy groups. The major difference appeared with the importance of narrative stories for the high hesitancy group. A study of 480 anti-vaccine websites in 2016 found that these websites utilized both trusted sources and anecdotal evidence(Moran et al., 2016).

The high hesitancy group, while hard to persuade and not as variable in their scores compared to the other groups, they still had relative preferences for more trusted sources, HPV, stories instead of statistics, and a hyperlink. Literature examining anti-vaccine sentiment point to the emotion of decision-making (Tomljenovic et al., 2020), where stories can elicit more emotion that large statistics. In a review of an anti-vaccine social networks, one network targeted parents who were undecided about vaccination and were pregnant for the first time or new mothers using maternal empowerment, natural solutions, and fear (Bradshaw et al., 2021). Over the next decade, unfortunately there will be more people telling stories of being affected by HPV related cancers who were vaccine eligible but who did not get it. Even the Centers for Disease Control and Prevention's website on cervical cancer has a similar story of a young woman who got cervical cancer and was not vaccinated (Centers for Disease Control and Prevention, 2024).

The differences in perceived persuasiveness by vaccine hesitancy in our sample could have been primed by what participants see in their real-world social media feeds, where those in the high-hesitancy groups may see more stories compared to statistics. In a study examining the persuasive tactics used in social media posts about the HPV vaccine, the authors found that both pro- and anti-vaccine articles frequently shared statistics, public health authority figures, links to other sources, scientific research, and scientific authority figures. In addition, the anti-vaccine messages used lawsuits, personal narratives, legal authority figures, medical authority figures, and scientific authority figures (McKenzie et al., 2024). Furthermore, around 40 % of Facebook posts from 2006 to 2016 represented HPV vaccine risk amplification, and those posts had significantly greater forward momentum, shares, and comments compared to pro-HPV vaccine posts (Luisi, 2021). As public health agencies and health communication professionals build social media posts towards targeted groups, these results can serve as a baseline in how to construct messages, such as making sure to include narrative stories as a part of the post when targeting vaccine-hesitant parents.

All of the groups in our study were more persuaded by posts for HPV over influenza vaccine and general adolescent vaccines. In the latest 2023 National Immunization Teen Survey, flu vaccine was 55.4 % compared to 76.8 % of adolescents receiving at least one HPV vaccine (Pingali, 2024). This may reflect national efforts increase HPV vaccine uptake though a variety of interventions aimed at multiple audiences (Reiter et al., 2018).

### 4.1 Limitations

Although these are national data, they are not representative nor was this a naturalistic study of actual social media use. However, this allowed us to test these posts in a controlled environment to the distilled dimensions. The next iterations of these types of studies could test bundled dimensions, such as posts with both narrative stories and statistics versus the narrative story alone or test bundled vaccine messages versus separate vaccine type messages in formulating social media posts aiming to increase vaccination rates. Limitations include the cross-sectional design (hindering ability to determine causality of associations) and limited racial diversity of the participants. Additionally, the study did not include perspectives from fathers/male identifying caregivers which may have differed.

## 5 Conclusion

These findings highlight the importance of messages from trusted organizations like the AAP, especially, when they include links to additional resources. Trusted sources enhance credibility, increasing the likelihood that parents will engage with the information about vaccination. For vaccine-hesitant individuals, our results suggest that sharing a personal stories or narrative-driven posts are more effective than statistical ones. This is not to say that only stories are effective in persuasion of vaccine-hesitant parents, but when posts were tested on between narrative and statistical dimension, these parents did perceive the posts with narrative messages as more persuasive. This approach fosters emotional connections and build trust, helping reduce resistance and promoting open dialogue about vaccination.

## CRediT authorship contribution statement

**Teresa M. Imburgia:** Writing – review & editing, Writing – original draft, Visualization, Validation, Formal analysis. **Holly B. Fontenot:** Writing – review & editing, Supervision, Resources, Project administration, Methodology, Funding acquisition. **Gary H.R. Glauberman:** Writing – review & editing, Supervision, Methodology, Data curation. **Erica Liebermann:** Writing – review & editing, Methodology, Conceptualization. **Melanie L. Kornides:** Writing – review & editing, Methodology, Conceptualization. **Eunjung Lim:** Writing – review & editing, Validation, Formal analysis, Conceptualization. **Masako Matsunaga:** Writing – review & editing, Validation, Methodology, Data curation. **Gregory D. Zimet:** Writing – review & editing, Supervision, Resources, Methodology, Funding acquisition, Formal analysis, Data curation, Conceptualization.

## Consent to participate

The University of Hawai'i at Manoa Institutional Review Board approved this study.

## Declaration of competing interest

Gregory Zimet has served as an external advisory board member for Moderna and Pfizer and as a consultant to Merck. In addition, Gregory Zimet and Holly Fontenot have received investigator-initiated research funding from Merck, administered through their universities. All other authors have no conflicts of interest or financial disclosures.

## Data Availability

Data will be made available on request.
